# PREPARE: PreoPerative Anxiety REduction. One-Year Feasibility RCT on a Brief Psychological Intervention for Pancreatic Cancer Patients Prior to Major Surgery

**DOI:** 10.3389/fpsyg.2020.00362

**Published:** 2020-03-05

**Authors:** Veronica Marinelli, Olivia Purnima Danzi, Maria Angela Mazzi, Erica Secchettin, Massimiliano Tuveri, Deborah Bonamini, Michela Rimondini, Roberto Salvia, Claudio Bassi, Lidia Del Piccolo

**Affiliations:** ^1^Department of Surgery, Dentistry, Pediatrics and Gynecology, University of Verona, Verona, Italy; ^2^Department of Neurosciences, Biomedicine and Movement Sciences, University of Verona, Verona, Italy; ^3^Pancreas Institute, University Hospital of Verona (AOUI), Verona, Italy

**Keywords:** preoperative anxiety, psychological intervention, pancreas surgery, feasibility RCT, self-efficacy

## Abstract

**Introduction:**

The aim of the present paper is to establish feasibility and required power of a one-session psychological intervention devoted to increasing patient’s self-efficacy and awareness in dealing with anxiety symptoms before major pancreatic cancer surgery.

**Methods:**

Parallel assignment RCT. All consenting patients listed for pancreatic major surgery during day-hospital visits (T0) between June 2017–June 2018 were assigned randomly in blocks of ten to a psychological intervention vs usual care group to be held the day before surgery (T1). The psychological intervention provided the patient the opportunity to increase self-efficacy in dealing with anxiety by talking with a psychologist about personal concerns and learning mindfulness based techniques to cope with anxiety.

**Results:**

400 patients were randomized into the experimental vs. usual care group. 49 and 65, respectively, completed baseline and post-intervention measures. The dropout rate between day-hospital (T0) and pre-surgery intervention (T1) was high (74.5%) due to several management and organization pitfalls. The main outcome, perceived self-efficacy in managing anxiety, showed a significant increase in the intervention group compared to the control group (*p* < 0.001), and was related to a reduction in state anxiety (*p* < 0.001). The intervention group perceived also lower emotional pain (*p* = 0.03). A power analysis was performed to define the appropriate sample size in a definitive RCT.

**Conclusion:**

Beneath the complexity in retaining patients along their trajectory in pancreatic surgery department, when they had the opportunity to follow a brief psychological intervention, most of them adhered, showing a significant reduction in preoperative emotional distress and less emotional pain perception after surgery. Even if results need caution because of the high attrition rate, we can infer that our psychological intervention has the potential to be proposed in surgical setting, being short, easy to learn and applicable to a wide range of patients.

**Clinical Trial Registration:**

The trial was registered on ClinicalTrials.gov (identifier: NCT03408002). The full protocol is available from the last author.

## Introduction

High levels of anxiety are common in patients who attend surgery (Guo, 2015) due to uncertainty, concerns and worries related to the potential physical and mental damage of surgery (Perks et al., 2009).

“Preoperative anxiety” can be considered a form of state anxiety and is defined as an unpleasant state of discomfort or tension related to the condition of waiting to undergo anesthesia and surgery (Maranets and Kain, 1999). Percentages reported in literature vary between 25 and 85%, according to the study (Norris and Baird, 1967; Mitchell, 2010, 2012; Mulugeta et al., 2018; Stamenkovic et al., 2018). Kindler et al. (2000) arranged the causes of preoperative anxiety into three dimensions: the fear of the unknown, the idea of being sick, and the possibility of life ending. This condition contributes to increasing the perception of worry, fear and uncertainty, which may be associated with depressive symptoms (Janis, 1958; Johnston, 1988; Miller et al., 1989; Guo, 2015) to the ability to cope with illness and to psychological resiliency (Johnston, 1988; Powell et al., 2016).

Anxiety levels have been found to be associated with factors related to the context, such as the organization of hospitalization and the degree of information provided; to patient’s psychosocial functioning in terms of cognitive style, behavioral and coping strategies (Krohne, 1989; Miller, 1996; Cohen and Taylor, 2002); and to the quality of social support perceived (Kulik and Mahler, 1989). All these stressors negatively impact perceived anxiety before surgery (Tsimopoulou et al., 2015; Powell et al., 2016) and perioperative outcomes (i.e. perceived pain, days of hospitalization, use of analgesic drugs, number of readmissions) after surgery (Norris and Baird, 1967; Kopp et al., 2003).

Pancreatic cancer is the fourth-leading cause of cancer-related death in Western countries (Rahib et al., 2014; Malvezzi et al., 2014, 2015; Siegel et al., 2015). This cancer is characterized by a very unfavorable prognosis, with a 5-year survival rate not exceeding 5%. The only way to cure the patient is surgical excision of the tumor (Ducreux et al., 2015; Masiak-Segit et al., 2018). Moreover, most patients with gastric and pancreatic cancer have advanced to an incurable stage at the time of diagnosis (Matsushita et al., 2005); therefore, only a small cohort of patients receive surgery, which is the only chance to obtain a prolonged survival. This condition increases psychological distress both because patients hope to recover in a condition of high insecurity and at the same time suffer all the stressors related to the uncertainty of major surgery (Matsushita et al., 2005). This explains why, perceived pain, anxiety and depression are particularly high in pancreatic patients when compared with other malignancies (Clark et al., 2010). Moreover, long-term postoperative morbidity can have a major impact on overall quality of life (Sun et al., 2016). Hence, pancreatic patients are particularly vulnerable and in need of an interdisciplinary approach to symptom and pre- and post-surgery management.

Despite widespread efforts in other cancer populations and surgical settings (Powell et al., 2016), no specific psychological intervention has been reported in the literature on pancreatic patients. To date, Sun et al. (2016) showed that a supportive care intervention based on comprehensive quality-of-life assessment (QOL), nurse-administered educational sessions and interdisciplinary care meetings were feasible and acceptable for pancreatic patients.

A recent meta-analysis (Powell et al., 2016), including 105 studies conducted between 1970 and 2014, reported different approaches to help patients reduce preoperative anxiety and stress before undergoing cardiology, orthopedic and abdominal surgery. The provision of information, relaxation techniques, sensory approaches, behavioral instructions, cognitive interventions, and emotion- and hypnosis-based techniques were generally described as effective in reducing postoperative pain, the length of the hospital stay (mean difference of 0.52 days) and negative affect (mainly assessed using anxiety scales). Tsimopoulou et al. (2015) reported in a systematic review that the most effective psychological interventions for stress management before cancer surgery (mainly breast, prostate, colorectal) were breathing, progressive muscle relaxation, meditation and mindfulness techniques, “guided imagery” (where participants were asked to imagine being at a safe and comfortable place), problem solving and coping strategies. Appropriate coping strategies, such as problem solving and emotion regulation contribute to enhance self-efficacy (Bandura, 1997a). Self-efficacy according to Bandura (1982) is a personal judgment of “how well one can execute a courses of action required to deal with prospective situations”. It strongly influences both the power a person actually has to face challenges competently and the choices a person is most likely to make (Luszczynska and Schwarzer, 2015). Literature reports that with increased self-efficacy, individuals show greater confidence in their ability and thus are more keen to adopt healthy behaviors (Luszczynska and Schwarzer, 2015). Four factors contribute to affect self-efficacy: the experience of mastery (success raises self-efficacy, while failure lowers it); modeling based on vicarious experience; social persuasion (i.e. motivational interview) and physiological factors (signs related to perceived anxiety). The last factor may be modified by using emotion regulation techniques, such as breathing, relaxation, meditation and mindfulness. Once the patient feels more confident in dealing with anxiety, also self-efficacy increases, contributing to enhance optimism, self-respect, internal control, achievement motivation and adaptation to the life changes (Jerusalem and Mittag, 1995). Conversely, the perception of inefficiency in controlling perturbing cognitions further increases the reactions to stress (Kent and Gibbons, 1987).

Therefore, given that pancreatic patients show a significant psychological burden related to their condition and that no specific psychological intervention has been described in the literature, despite the fact that it seems compelling and acceptable, we decided to plan a short psychological consultation mainly based on emotion regulation techniques, whose characteristics and feasibility will be described in this paper.

Specifically, the study aimed to achieve the following: (1) to obtain a general description of psycho-social variables in pancreatic patients by enrolling all consecutive patients admitted to surgery during 1 year; (2) test the acceptability (proportion of patients who agreed to participate in the study during its different phases) and the feasibility (recruitment rate and analysis of the causes for dropout taking into account the complexity of the clinical setting organization) of a psychological intervention for patients listed for pancreatic major surgery; (3) test if the psychological intervention contributes to increase perceived self-efficacy (primary outcome) with a concurrent reduction in state anxiety; (4) to test the effect of the intervention on perceived pain, length of hospital stay and number of postoperative complications within 30 days, after surgery (secondary outcomes); (5) collect primary outcome data to determine the sample size required for a definitive RCT (power analysis).

## Materials and Methods

### Study Design

We conducted a two-arm parallel randomized feasibility study of a one-session manualized psychological intervention versus a “treatment as usual” control for patients undergoing major pancreatic surgery.

### Setting

The study took place at the Pancreas Institute of the University Hospital of Verona (AOUI), Italy, which is the first multidisciplinary high-volume (more than 450 resections per year) Italian center entirely dedicated to diagnosis, treatment and research in the field of pancreatic diseases. The Pancreas Institute is one of the most important international centers for pancreatic surgery, and patients come from all over Italy.

### Participants

The inclusion criteria were: 18–80 years old, cognitively able to give signed informed consent to participate in the study and being scheduled to have general anesthesia for major pancreatic surgery. Exclusion criteria were: unable to understand Italian or postponing, modifying or canceling surgery.

Patients were identified when they were attending the counseling session (day-hospital) with a surgeon and an anesthesiologist to receive Computed tomography angiography, blood exams, Electro and Echo-cardiogram. The major topic of these consultations was to evaluate ASA (American Society of Anesthesiologists) conditions (Knuf et al., 2018) to undergo surgery, to discuss the medical procedure and its risks and to explain the informed consent to be signed on the surgery sheet. Eligible patients were asked to participate in the study after this evaluation.

### Description of the Psychological Intervention

Following Bandura’s (1997b) claim that both the experience of mastery and physiological factors may contribute to modify self-efficacy perception, we proposed to the intervention group a brief psychological consultation that aimed to:

(1) help patients express their concerns related to surgery and to learn simple techniques based on body awareness and related imagery techniques;
(2) be applicable in a very heterogeneous population of patients who widely differed in terms of age, psychological needs (e.g. emotional distress) and resources (e.g. metacognitive abilities);
(3) be brief and easy to propose. This because often in surgical setting patients have no opportunity to see a psychologist several times before surgery.

To address these aims we implemented a one-session psychological consultation lasting 1-h. It was organized into two parts: (1) an initial phase in which the patient was invited by the clinical psychologist (VM) to disclose her concerns and worries about surgery, following the protocol of Svensson et al. (2016), with the purpose of promoting the expression and identification of the patient’s emotional state and to favor therapeutic alliance; and (2) a second phase in which the “Four Elements” protocol for stress management proposed by Shapiro (2009) was applied to reduce anxiety and foster patients’ abilities to cope with stress. The sequence of the four elements (earth, air, water, fire) proposed by Shapiro was selected because it is easy to remember. Moreover, it refers to mindfulness based techniques (Weick and Putnam, 2006) such as grounding (earth element), breathing (air), and “guided imagery” (water element was connected with the sensation of suckking something inducing salivation, such as a lemon; in fire image, participants were asked to imagine being at a safe and comfortable place and describing it in detail). These techniques have been shown to be learnable, useful (Liu et al., 2015) and to increase self-efficacy by reducing perceived stress (Firth et al., 2019). Mindfulness improves the psychological and physical symptoms of anxiety through relaxation and by helping the individual to become aware of what occurs internally in each moment, focusing on his/her positive, negative and neutral experiences by reducing judgmental attitude (Germer et al., 2005). It has also been shown to enhance emotion regulation (Tang and Leve, 2016).

**FIGURE 1:**
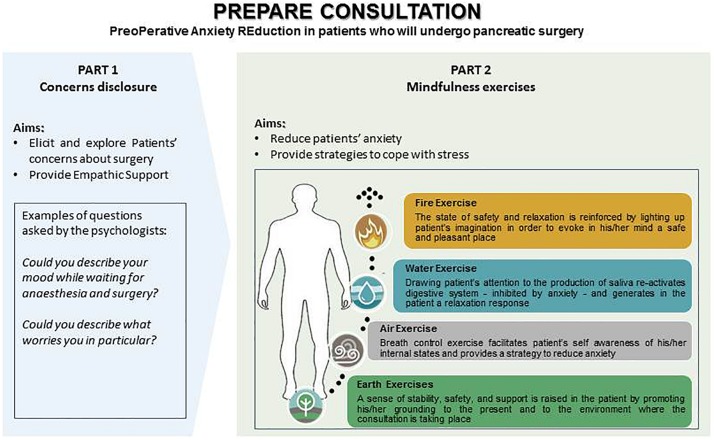
Description of the psychological intervention adapted from Svensson et al. (2016) and the “Four Elements protocol” proposed by Shapiro (2009).

Subjects tried these techniques together with the clinical psychologist and shared with her the feelings, emotions and sensations they perceived. The specific features of the psychological intervention protocol are summarized in Figure 1.

At the end of the consultation, patients were provided with a red stamp to be applied on the identification bracelet, which acted as a reminder to practice the exercise during moments of greater stress. The red stamp was intended to anchor patients on positive reinforcement (Skinner, 1954) and self-awareness (Kabat-Zinn, 1991).

The psychological intervention was manualized, and the clinical psychologist who had to provide the intervention was trained and checked for adherence to the protocol by the principal investigator (LDP) by asking to the psychologist to replicate the intervention under direct observation, with several patients, until it was easily and correctly run.

The control group received usual care, that is no specific intervention to deal with pre-surgical anxiety. Patients could talk informally with the clinical psychologist if they desired but no specific intervention was allocated.

### Outcomes

(1) To obtain a baseline description of psycho-social variables in pancreatic patients, self-administered sociodemographic and clinical questionnaires were collected when patients came to day-hospital for pre-surgery visit (T0), an average of 1 month before surgery: STAI-Y2 (Spielberger, 1983b; Pedrabissi and Santinello, 1989), *PHQ-9* (Spitzer et al., 1999), *GSES* (Sibilia et al., 1995), *MSPSS* (Prezza and Santinello, 2002), *FACT-G* (Cella et al., 1993), *FACIT F* (Cella et al., 1993), *BRIEF COPE* (Carver et al., 1989). See Table 1 for a detailed description of the instruments. A research psychologist (OPD) with clinical competence helped those who required support to complete the questionnaires.

**TABLE 1:** List of questionnaires and variables collected at each stage of the study.

Instruments (Author; Italian adaptation)	*No.* of items	Type of scale (anchoring scores)	Clinical domain or type of information collected	Cut-off/Range	Scoring	T0	T1	T2	T3
State-trait anxiety inventory STAY-Y2 Spielberger, 1983a; Pedrabissi and Santinello, 1989	20 items	4 point likert scale (1 never – 4 very often)	Trait anxiety	40	Sum of all items Reverse items 1,3,6,7,10,13, 14,16,19	X			
Patient health questionnaire PHQ-9 Spitzer et al., 1999	10 items	4 point likert scale (0 never – 3 about every day)	Presence of depressive symptoms	10	Sum of all items	X			
General self efficacy scale GSES Sibilia et al., 1995	10 items	4 point likert scale (1 completely agree – 4 completely disagree)	Self-efficacy perception	10—0	Sum of all items	X			
Multidimensional scale of perceived social support MSPSS Prezza and Santinello, 2002	12 items	4 point likert scale (1 completely disagree – 6 completely agree)	Social Support	12–84	Sum of all items	X			
Functional assessment of cancer therapy – general (FACT-G) Cella et al., 1993	27 items	4 point likert scale (1 little – 4 very)	Assessment of cancer therapy	27–108	Sum of all items	X			
Functional assessment of chronic illness therapy-fatigue (FACT-F) Cella et al., 1993	12 items	4 point likert scale (1 anything – 4 very)	Cancer related fatigue	0–52	Sum of individual item scores^∗^13/Number of items answered	X			
The brief coping orientation to problems experienced (COPE) Carver et al., 1989	28 items	4 point likert scale (1 I don’t usually do this – 4 I usually do this)	Coping Styles	28–112	Sum of all items	X			
Amsterdam Preoperative Anxiety and information scale (APAIS) Buonanno et al., 2017	6 items	6 point likert scale (1 for nothing – 6 very much)	Pre-surgical anxiety	14	Sum of all items		X		
State-trait anxiety inventory STAY-Y1 Spielberger, 1983a; Pedrabissi and Santinello, 1989	20 items	4 point likert scale (1 never – 4 very often)	State anxiety	40	Sum of all items Reverse items 1,2,5,8,10,11, 15,16,19,20		X	X	
Visual analog scale for self-efficacy	1 item	10-point visual analog scale (0 very low – 10 high)	Perceived self-efficacy in managing anxiety	Continuous scale			X	X	
Brief pain inventory (BPI-I) Caraceni et al., 1996	16 items	Specific questions on pain and 12 10-point visual analog scales (0 no pain–10 worse pain)	quality and intensity of physical pain	Continuous scale	Sum of all items				X
Visual analogue scale for pain (VAS-P) Huskisson, 1974	1 item	10-point visual analog scale (0 no pain – 10 worse pain)	Perceived pain	Continuous scale					X
Length of stay			Number of days of hospitalization collected on hospital register						X
Number of complications		Surgeons classification	Number of complications						X

(2) For the second aim, the following feasibility measures were considered:
  (a) The proportion of patients meeting inclusion criteria, who agreed to participate in the study during pre-surgical counseling and the proportion of patients who agreed to undergo psychological treatment the day before surgery (T1). At T1, once admitted, eligible patients were enrolled by the clinical psychologist of the surgery department (VM), who asked them to report on a 10-point Likert scale their perceived self-efficacy in managing anxiety and to complete the Amsterdam Preoperative Anxiety and Information Scale (APAIS) (Buonanno et al., 2017) and the Spielberger state anxiety scale [*STAI-Y1* (Spielberger, 1983a, b; Pedrabissi and Santinello, 1989)].
  (b) The recruitment rate during the different phases of the study, indicating causes for dropout, by collecting clinical register data.
(3) To test the effect of the psychological intervention on perceived self-efficacy (primary outcome), those who underwent the psychological intervention were asked to fulfill the 10-point Likert scale on perceived self-efficacy in managing anxiety within 1 h after the psychological intervention (T2). Usual care group did not complete any questionnaire, as it was supposed that with no treatment, no change in self-efficacy and state anxiety could be observed; moreover, they would find strange to answer the same questions posed only 1 h before with no changes in their activity.
  To verify if the psychological intervention had an effect in reducing state anxiety the Spielberger state-anxiety [*STAY-Y1* (Spielberger, 1983b; Pedrabissi and Santinello, 1989)] was also administered within 1 h after the psychological intervention (T2).
(4) Secondary outcomes were collected after surgery (T3): quality and intensity of physical perceived pain using *BPI* (Caraceni et al., 1996) and *VAS* (Huskisson, 1974) between the 3rd and 7th day after surgery; length of hospital stay and the frequency of postoperative complications within 30 days were gathered using the clinical register of the Pancreas Institute.
(5) The primary outcome data distribution was considered to calculate the sample size required for a definitive RCT (power analysis).

### Changes to Protocol Measurements

The original protocol reported that for a subgroup of patients in the experimental group, the following psycho-physiological parameters would be measured:

• Skin Conductance Reactivity (SCR) both in the patient and the psychotherapist
• Heart rate (HR) of the patient only

The aim was to analyze the trend of patient’s physiological arousal and, in conjunction with the therapist, to evaluate the quality of the therapeutic alliance, which would also be measured by Working Alliance Inventory (WAI-C for the patient and WAI-T for the psychotherapist) (Horvath and Greenberg, 1989).

Inclusion criteria were: the absence of jaundice, feverish states, pancreatic pain with analgesic therapy, new neo-adjuvant therapy, drug therapies based on Benzodiazepines for anxiolytic purposes and cardio-vascular problems. Only very few patients of the experimental group satisfied these inclusion criteria, thus it was not possible to carry on this part of the study.

**FIGURE 2:**
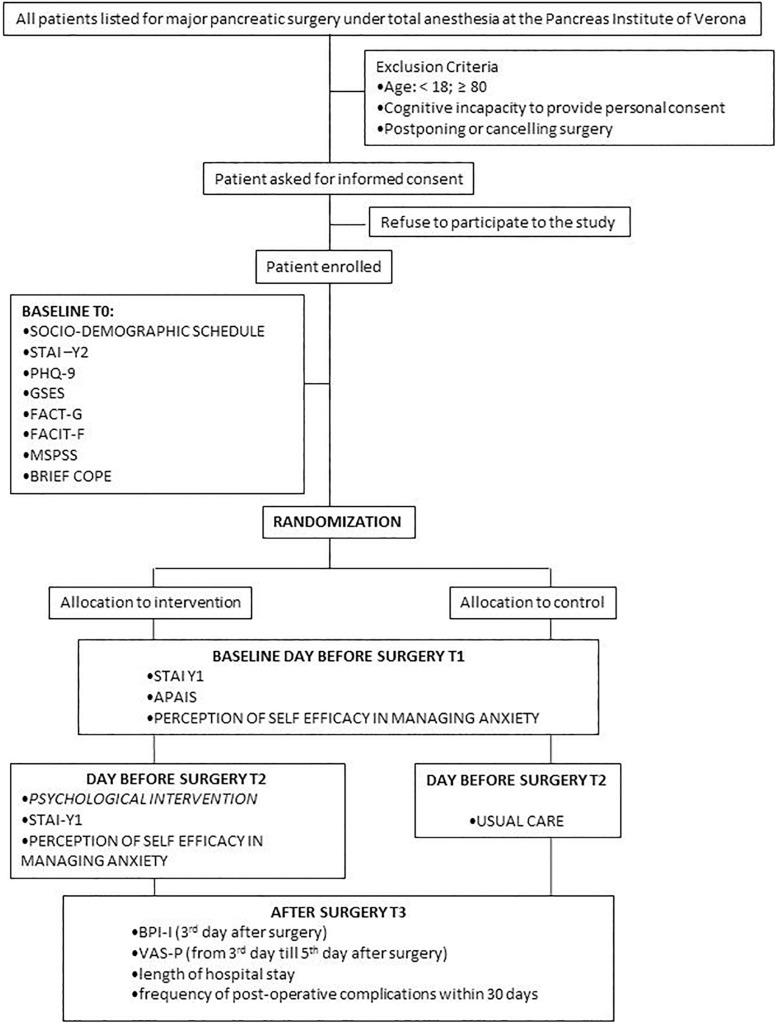
Flow chart of the study design.

The final trial profile following CONSORT guidelines (Eldridge et al., 2016) is reported in Figure 2.

### Sample Size

The clinical register of the Pancreas Institute reported that in 2016, 366 pancreatic resections were performed. Based on this information, to determine the sample size, we planned to collect data from mid-June 2017 to mid-June 2018, expecting to reach a final sample of approximately 400 patients listed for major surgery.

### Randomization

Eligible consenting participants were randomly assigned to two arms with equal allocation (on a 1:1 ratio). The list of randomization was computer generated by the research support office of the AOUI of Verona with the statistical software STATA 11 (StataCorp, 2009), using block randomization with a block size of 10. The name of the patients who were admitted to the hospital to undergo surgery (generally the day before) were communicated day by day to the researcher (OPD) who had allocated patients on the basis of the list of randomization at T0. The researcher then indicated to the psychotherapist (VM) which treatment she had to apply each day, on the basis of the list of allocation.

### Statistical Methods

In order to explore differences among the subsamples in terms of socio-demo and clinical characteristics we performed a set of bivariate comparisons, by using Chi2 test and Student’s *t*-test for independent groups (completers vs drop-out patients and “intervention” vs “usual care” patients).

Paired *t*-test was adopted to verify the change in perceived self-efficacy using a per-protocol approach. Pearson correlation was calculated to test the association between state anxiety reduction and self-efficacy increase.

The sample size required for a definitive RCT was calculated on the primary outcome data distribution, on the basis of a *t*-test for independent groups, assuming the absence of confounding effects (homogeneous groups).

Analyses were performed with Stata 15.1.

## Results

### Baseline Description of Psycho-Social Variables in Pancreatic Patients

**FIGURE 3:**
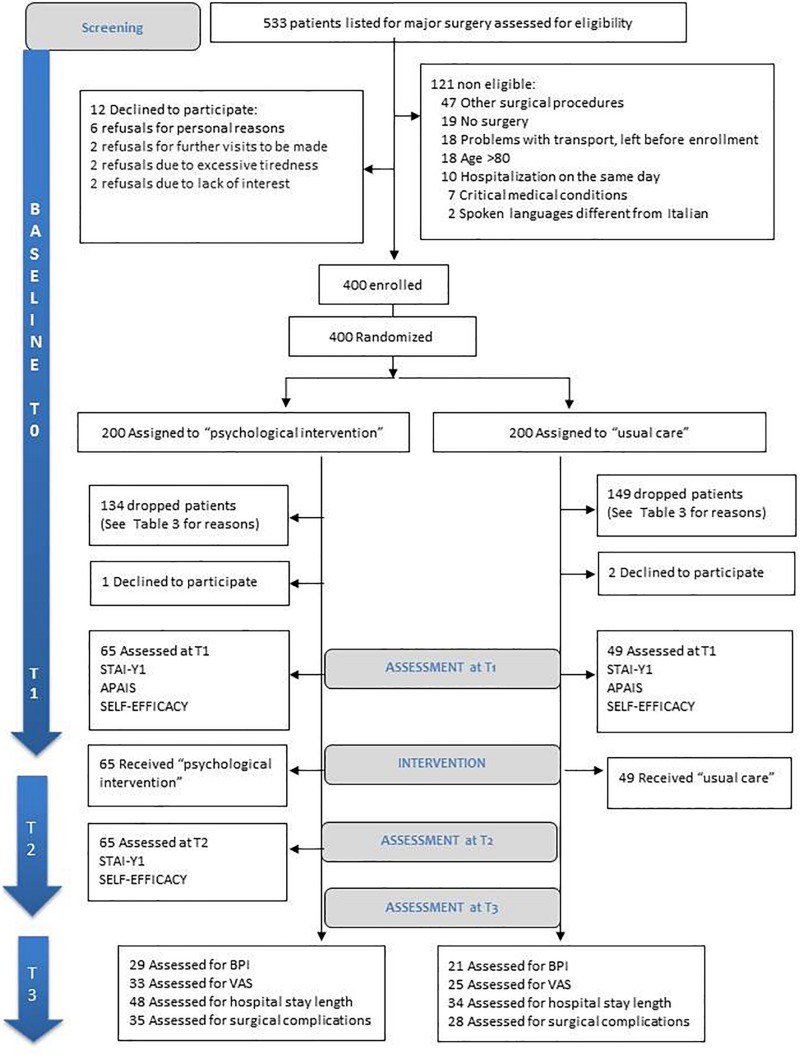
CONSORT (Eldridge et al., 2016) diagram of patient recruitment.

Figure 3 reports participants flow.

A total of 533 patients were screened prior to eligibility assessment, 121 (22.7%) were excluded and 12 patients declined to participate (3% of eligible patients). The remaining 400 were recruited and randomly assigned to the intervention (*n* = 200) and control (*n* = 200) groups.

The baseline (T0) sociodemographic and clinical characteristics of the original sample of 400 patients (212 male patients, 53%) are shown in Table 2.

**TABLE 2:** Socio-demographic and clinical characteristics of patients included in the study.

	To total sample
Socio-demographic variables	*N* = 400
	*N*.(%)
**Gender**	
Male	212 (53)
**Age**	
<50	62 (16)
51–69	210 (53)
>70	122 (31)
**Education level**	
Until primary school	84 (21)
Middle school	10 (25)
High school	141 (35)
Degree	74 (19)
**Marital status**	
Married/Cohabitant	316 (79)
Divorced Widower	62 (16)
Unmarried	31 (8)
**Children**	
Yes	338 (85)
**Employment status**	
Student/Worker	160 (40)
Jobless	16 (4)
Housewife	38 (10)
Retired	186 (46)
**Citizenship**	
Italian	395 (99)
**Region coming from**	
Veneto	84 (21)
Smoke	
No	334 (84)
**Alcohol**	
No	396 (99)
**Psychotropic drugs use**	
Sleeping pills	35 (9)
Anxiolytics	34 (9)
Antidepressants	16 (4)
**Clinical variables**	**Mean (sd)**
PHQ-9	5.3 (4.8)
GSES	34.9 (5.2)
MSPSS	6.4 (0.9)
FACIT-F	41.8 (11.6)
FACT-G	55.3 (9.3)
Brief COPE	66.7 (9.5)
STAI-Y2	32.6 (9.8)

The mean age was 62 years. Most patients were married or engaged in a relationship (79%). A total of 54% had a high school diploma or degree. A total of 46.5% were retired from work, and only 4% were jobless. Approximately 79% came from Italian regions other than Veneto, which means they lived far from the hospital. Regarding clinical variables, 20% of patients exceeded the cut-off threshold for trait anxiety and 18% for depression symptoms (see Table 1 for cut-off references). Most of the patients had high social support and coping abilities. Eighty-five patients out of 400 (21%) used psychotropic drugs: 35 took sleeping pills (which is quite typical of the elderly and can be done for several reasons), 34 used anxiolytics (8 of whom used them in association with antidepressants) and 8 used antidepressants only.

### Feasibility Measures

At T1 (Figure 3), 134 patients in the intervention group (67%) and 149 in the control group (74.5%) dropped the study. Dropout reasons are presented in Table 3.

**TABLE 3:** Reasons for drop-out at T1 (*N* = 283).

	Intervention group	“Usual care” group	Total
Reason for drop-out	*N* = 134 (47.3%)	*N* = 149 (52.7%)	*N* = 283 (100%)
Hospitalization after 5 p.m. when the clinical psychologist that received patients was absent	40 (29.9%)	32 (21.5%)	72 (25.4%)
Hospitalization during festivity days: weekends or holidays when the clinical psychologist was absent	28 (20.9%)	40 (26.9%)	68 (24.0%)
Hospitalization when the clinical psychologist was not available having other clinical duties	19 (14.2%)	17 (11.4%)	36 (12.7%)
Changes in surgery planning (hospitalization the same day of surgery, surgery postponed or moved in a different hospital)	2 (1.5%)	7 (4.7%)	9 (3.2%)
Patient not available (for clinical reasons or because attending other clinical examinations)	8 (6.0%)	6 (4.0%)	14 (5.0%)
Patient did not undergo surgery	36 (26.9%)	40 (26.8%)	76 (26.9%)
Included by mistake (not satisfying inclusion criteria)	1 (0.7%)	7 (4.7%)	8 (2.8%)

Attrition was mainly due to organizational and logistic aspects (several patients were admitted to the hospital during weekends or evening hours, when the clinical psychologist of the surgery department, who received patients and provided the psychological intervention on allocation basis, was not available) or because patients were not able to undergo surgery for different reasons. Statistical comparison showed that the reasons for dropout were equally distributed into the two groups (χ^2^ = 10.13, dof = 6, *p* = 0.12).

The comparison between completers and patients who dropped-out is presented in Table 4. Differences were observed for employment status (χ^2^ = 10.6, *p* = 0.01), smoking habits (χ^2^ = 5.28, *p* = 0.02), PHQ-9 (*t* = 2.6, *p* < 0.01) and FACT-G (*t* = 2.06, *p* = 0.04). All other variables were comparable. Of the 117 remaining patients at T1, one in the intervention group and two in the control group declined to complete the questionnaires. A total of 114 patients were finally assessed the day before surgery (Figure 3). Of these patients, 54% were over the cut-off for state anxiety (STAY-Y1, Spielberger, 1983b; Pedrabissi and Santinello, 1989), and 55% for pre-surgical anxiety [APAIS Buonanno et al., 2017)], with no difference between the experimental and the control group. Additionally, the comparisons of sociodemographic and clinical variables showed that the control and the intervention groups remained balanced after losing approximately 70% of randomized subjects (Table 5).

**TABLE 4:** Socio-demographic and clinical variables between completers (*n* = 114) and dropouts (*n* = 284).

Socio-demographic	Completers	Drop-out	
variables	(*n* = 114)	(*n* = 384)	Comparison
	*N*.(%) < /cps:bf >	*N*.(%) < /cps:bf >	Chi^2^(*p*−value)
**Gender**			2.95 (0.09)
Male	53 (46.5)	159 (55.9)	
**Age**			3.91 (0.14)
<50	13 (11.4)	49 (17.5)	
51–69	69 (60.5)	141 (50.4)	
>70	32 (28.1)	90 (32.1)	
**Education level**			0.22 (0.97)
Until Primary school	22 (19.3)	60 (21.1)	
Middle school	29 (25.4)	72 (25.4)	
High school	42 (36.9)	99 (34.9)	
Degree	21 (18.4)	53 (18.7)	
**Marital Status**			0.83 (0.66)
Married/Cohabitant	93 (81.6)	221 (77.8)	
Divorced Widower	14 (12.3)	39 (13.7)	
Unmarried	7 (6.1)	24 (8.5)	
**Children**			0.71 (0.39)
Yes	99 (86.8)	237 (83.5)	
**Employment status**			10.6(0.01)*
Student/Worker	41 (35.9)	119 (41.9)	
Jobless	2 (1.8)	12 (4.2)	
Housewife	19 (16.7)	19 (6.7)	
Retired	52 (45.6)	134 (47.2)	
**Citizenship**			0.31 (0.57)
Italian	112 (98.3)	281 (98.9)	
**Region coming from**			0.31 (0.58)
Veneto	22 (19.3)	62 (21.8)	
**Smoke**			5.28(0.02)*
No	103 (90.4)	229 (80.9)	
**Alcohol**			0.02 (0.87)
No	113 (99.1)	281 (98.9)	
**Psychotropic drugs use**			***Z*(*p*−value)**
Sleeping pills	8 (7)	27 (9.4)	0.60 (0.44)
Anxiolytics	8 (7)	26 (9.1)	0.45 (0.50)
Antidepressants	4 (3.5)	12 (4.2)	0.10 (0.75)

**Clinical variables**	**Mean(sd)**	**Mean(sd)**	***t*−test(*p*−value)**

STAI-Y2	31.4 (9.9)	33.1 (9.9)	1.6 (0.12)
PHQ-9	4.4 (4)	5.7 (4.9)	2.6(0.00)**
GSES	34.9 (5.1)	34.9 (5.2)	0.22 (0.82)
MSPSS	6.5 (0.81)	6.3 (0.9)	1.8 (0.07)
FACIT-F	43.1 (10.2)	41.3 (12.1)	1.54 (0.12)
FACT-G	89.3 (13.9)	85.8 (17.2)	2.06(0.04)*
Brief COPE	67.2 (8.8)	66.7 (9.6)	0.53 (0.59)

*p < 0.05, **p < 0.01.

**TABLE 5:** Socio-demographic and clinical characteristics of patients distinguished by intervention and “usual care” group at T1 (day before surgery).

Socio-demographic	T1 Intervention	T1 “usual care”	Comparison
variables	group *N* = 65	group *N* = 49	at T1
	*N*.(%)	*N*.(%)	Chi^2^(*p*−value)
**Gender**			0.21 (0.64)
Male	29 (45)	24 (49)	
**Age**			1.02 (0.60)
<50	9 (14)	4 (8)	
51–69	38 (58)	32 (65)	
>70	18 (28)	13 (27)	
**Education level**			1.98 (0.58)
Until Primary school	12 (19)	9 (18)	
Middle school	20 (31)	10 (20)	
High school	23 (35)	19 (39)	
Degree	10 (15)	11 (22)	
**Marital status**			2.13 (0.71)
Married/Cohabitant	53 (82)	40 (82)	
Divorced Widower	8 (12)	6 (12)	
Unmarried	4 (6)	3 (6)	
**Children**			0.09 (0.76)
Yes	57 (88)	42 (86)	
**Employment status**			1.89 (0.22)
Student/Worker	26 (40)	16 (32)	
Jobless	3 (5)	0 (0)	
Housewife	10 (15)	9 (18)	
Retired	36 (40)	24 (49)	
**Citizenship**			2.70 (0.10)
Italian	65 (100)	47 (96)	
**Region coming from**			00 (0.99)
Veneto	12 (19)	9 (18)	
**Smoke**			0.03 (0.86)
No	59 (91)	44 (90)	
**Alcohol**			1.33 (0.25)
No	65 (100)	48 (98)	
**Psychotropic drugs use**			***Z*(*p*−value)**
Sleeping pills	3 (5)	4 (8)	0.78 (0.43)
Anxiolytics	2 (3)	5 (10)	1.57 (0.12)
Antidepressants	2 (3)	2 (4)	0.29 (0.77)

**Clinical variables**	**Mean (sd)**	**Mean (sd)**	***t*-test (*p*-value) **

PHQ-9	4.6 (4.2)	4.2 (3.6)	0.58 (0.56)
GSES	35.0 (5.0)	34.5 (5.4)	0.43 (0.66)
MSPSS	6.5 (0.9)	6.6 (0.7)	0.90 (0.37)
FACIT-F	2.7 (10.5)	43.6 (9.9)	0.46 (0.65)
FACT-G	55.5 (10.0)	56.7 (7.0)	0.72 (0.47)
Brief COPE	67.1 (9.7)	66.9 (8.7)	0.07 (0.94)
STAI-Y2	31.3 (8.4)	32.1 (10.6)	0.49 (0.62)
APAIS	14.9 (6.2)	15.3 (6.9)	0.13 (0.90)
STAI-Y1	43.1 (13.7)	43.4 (12.1)	0.38 (0.70)
EFFICACY	6.9 (1.7)	7.1 (2.1)	0.58 (0.57)

### Effect of the Psychological Intervention on Perceived Self-Efficacy

At T2, the comparison after psychological intervention in the intervention vs usual care group showed a significant increase (7.1 vs 8.3; *t* = 3.4, *p* < 0.01) in the average scores of perceived self-efficacy in managing preoperative anxiety.

As an effect of the psychological intervention, we observed a significant decrease in state anxiety within the intervention group (STAI-Y1) (43.4 vs 28.2; *t* = 7.5, *p* < 0.01), which significantly correlated with an increase of perceived-self-efficacy (*r* = 0.51, *p* < 0.01).

### Secondary Outcomes Collected After Surgery

At T3, several patients were in the intensive care unit or discharged early from the hospital; therefore, data on pain perception were collected only on 51% of the patients who participated in the trial. Similarly, the frequency of surgical complications within 30 days was collected in 55.3% of the patients, and the length of stay was present in clinical records for 72% of the patients. The emotional component of pain on BPI showed a significant decrease in the intervention group compared to usual care (*d* = 1.4; *p* = 0.02). No significant difference was found in the length of hospital stays [usual care = 13.6 (14.1); intervention group = 12.5 (12.0); *p* = 0.62] and for surgical complications (Table 6).

**TABLE 6:** Secondary outcomes: measures collected after surgical intervention.

	Intervention group			“Usual care” group			Comparison	
Outcomes	*n*	Mean (sd)	95% CI	*n*	Mean (sd)	(CI)	*t*/*z*-test	*p*-value
BPI-physical pain	29	4.3 (1.6)	3.7–4.9	22	4.8 (2.3)	3.8–5.8	0.98	0.33
BPI-emotional	29	2.5 (1.8)	1.8–3.2	22	3.9 (2.4)	2.8–5.0	2.35	**0.02**
BPI-operative	29	4.5 (2.3)	3.6–5.3	22	5.3 (2.5)	4.2–6.4	1.25	0.22
Presence of surgical complications	21	47.7%	33.0–62.5	19	55.9%	39.2–72.6	0.51	0.48
Length of stay	58	12.5 (12.0)	9.8–15.2	46	13.6 (14.1)	9.4–17.8	0.49	0.62

*Bold values means significant values.*

### Sample Size Required for a Definitive RCT

**FIGURE 4:**
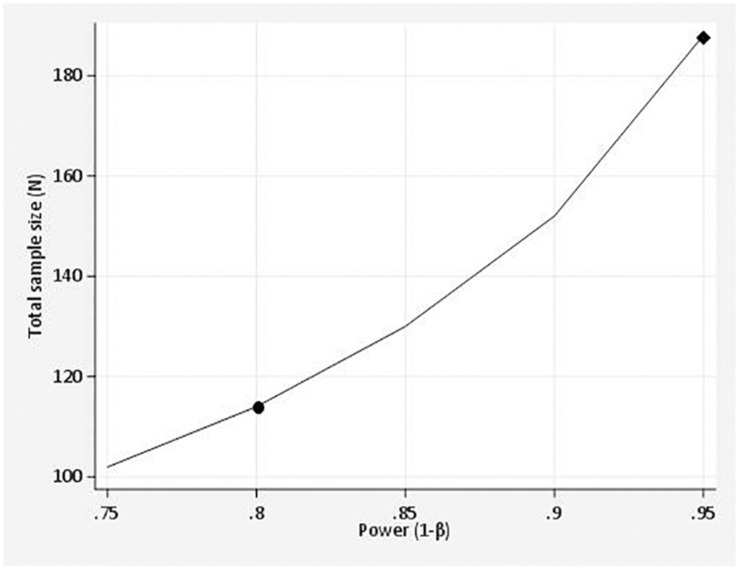
Power curve shows the relationship between the power and the sample size of a student’s *t*-test for independent groups: the point on the curve, marked with a circle, represents the 80% power and the sample size of 114 participants. This means that at least 57 patients in each group are needed to detect a different of 1 point on the self efficacy Likert scale, given a power of 80%. The participants patients might increase to 188, if we want to reach a power of 95% (see diamond on the power curve). The asumed parameter values include: alpha level (5%), mean value (7), the standard deviation (1,9) and the effect size (1).

Finally, a statistical power analysis was performed in order to get the sample size needed to detect a clinically meaningful significance of at least 1 point on the self-efficacy scale. The one point minimal change was set on the basis of Hawker et al. (2011) paper on numerical scales. A sample size of at least 57 patients in each group is needed, to obtain in), given a power of 80%, a (two-tailed) significance level of 5%, and assuming, on the basis of observed data at T1, that the frequency distribution of perceived self-efficacy is normally distributed, with a mean value of 7 points and a standard deviation of 1.9. This number might increase to 94 if we want to reach a power of 95% (see Figure 4).

## Discussion

Pancreatic patients show a significant psychological burden related to their condition and to preoperative anxiety, which tend to remain over all symptom trajectory (Hallet et al., 2019). Our study investigated the feasibility of a one-session psychological intervention devoted to increase patient’s self-efficacy and awareness in dealing with anxiety symptoms, by using emotional support and mindfulness techniques. Literature already reports that mindfulness showed some promising results for increasing self-efficacy and its positive effect on cancer patients (Branstrom et al., 2010; Sanaei et al., 2014; Carlson, 2018). Additionally, based on Powell et al. (2016) review, positive secondary outcomes were expected for pain perception, number of compliances and length of hospital stay.

### Study Limitations

The recruitment rate between day hospital (T0) and the day before surgery (T1) was poor and only 28.5% of patients enrolled at T0 were able to complete T1 questionnaires and to be allocated to the intervention or control groups. The randomization of patients done during day-hospital stay was the main reason for drop-out. Several patients did to arrive to surgery after day-hospital evaluations (19%), or were hospitalized during the weekend or after 5 p.m., when the clinical psychologist who received patients was absent or was busy with other clinical duties (44%). Retained patients who were part of the RCT were comparable to the initial sample for the majority of clinical and sociodemographic characteristics, with the exclusion of employment status, smoking habits, depressive symptoms (PHQ-9 score) and Functional Assessment of Cancer Therapy. Additionally, after surgery (T3), 49% of patients were lost because they were in the intensive care unit or discharged early from the hospital. Therefore, primary and secondary outcomes need to be considered with caution.

### Generalizability

The difficulty of applying a psychological intervention in our study was that all patients were admitted to the hospital just the day before surgery and had to adapt to the upcoming operation very quickly. Therefore, the psychological intervention had to appropriately help patients meet this need. Data in our sample confirm the utility to intervene near the surgery: if at baseline (T0) 20% of patients showed clinically significant rates of trait anxiety, the day before surgery (T1), more than half of the included patients had a score over the threshold in state anxiety (54%) and pre-surgical anxiety (55%), providing evidence that a psychological intervention is important and useful for these patients during this specific timeframe. Indeed, percentages reported in literature for patients admitted to hospital for surgery vary between 25 and 80% (Stamenkovic et al., 2018), depending on sociodemographic factors (gender and age), psycho-social variables (i.e. stress tolerance, coping strategies, concern about family, social support), type of surgery and anesthesia (general or local), history of prior surgery and cancer. Studies reporting data from patients attending different types of surgery account percentages around 60% (Norris and Baird, 1967; Mulugeta et al., 2018). Higher percentages (85%) were reported by Mitchell (2010, 2012) who collected data only from respondents by e-mail 24–48 h after surgery; Bedaso and Ayalew (2019) indicated a prevalence of 47% for patients scheduled for elective surgery in Ethiopia. Our sample showed that more than one patient every two had a level of anxiety over the threshold, which is in between data reported in other studies. On one side our sample of patients had a condition of high vulnerability and frailty, on the other, they showed good family support and high general self-efficacy perception, which both contribute to decrease anxiety (Krohne, 1989; Kulik and Mahler, 1989; Miller, 1996; Cohen and Taylor, 2002; Karanci and Dirik, 2003).

As far as regards acceptance of the psychological intervention, based on enrolment at baseline, only 3% of eligible patients declined to participate and also at T1, of the 117 remaining patients after drop-out, only one in the intervention group and two in the control group declined to complete the questionnaires, demonstrating that a brief psychological intervention is positively viewed and accepted by pancreatic patients. As far as regards the control group, most of the patients stayed with their family. The psychologist guaranteed informal support when detecting vulnerable patients or families and followed all of them immediately after surgery. Additionally, after surgery most of the patients in the intervention group reported anecdotally to the psychologist that they applied the psychological techniques at least once between the time of the psychological intervention and the surgery, finding them useful and easy to apply.

The distribution score of the main outcome variable “perceived self-efficacy” showed that the psychological intervention was effective in increasing the perceived ability to deal with anxiety before surgery and that this same numeric rating scale is useful, easy to apply and sensitive to change, as suggested by Hawker et al. (2011) for pain numeric rating scales. We also observed the correlation between the reduction in state anxiety and the increase in self-efficacy perception, confirming Bandura’s model (Bandura, 1982).

Regarding post-surgery outcome measures, we observed a positive trend in the intervention group, who reported lower emotional correlates of pain, as already shown by Cheung et al. (2003) and Theunissen et al. (2012). Previous literature showed that anxiety and depression were moderators of hospital length of stay and post-surgical complications (Halvorson et al., 1986; Dao et al., 2011; Stundner et al., 2013; Poole et al., 2016; Powell et al., 2016). In our study, no significant difference was observed in hospitalization length, which is indeed a variable connected to the complexity of the surgical procedures that these patients must undergo (Egawa et al., 2008). The variability in hospitalization length was also very high, contributing to a lower probability of significant differences.

### Implications for Progression From Feasibility to Future Definitive Trial

Future clinical trials aimed to confirm the results of our study should pay careful attention in addressing several management and organization pitfalls that might determine high attrition rates due to the peculiar organizational challenges characterizing a clinic that attracts several patients living far from the hospital and admitted during non-standard working times (weekend and evening). More in detail, we suggest that to overcome the attrition rate during pre-surgery data collection, it would be useful to involve more personnel (e.g. nurses) in receiving patients and administering psychological tests and to form more personnel capable to carry out the psychological intervention being available in different moments of the day. Another option would be to try to provide home-delivered support or online training of the psychological intervention, even if several patients were old and we were not so sure on their familiarity with the use of online devices and on the usefulness of a virtual intervention lacking interpersonal relationship.

To overcome post-surgical drop-out, we suggest fostering the cooperation of intensive care personnel through the use of a standard protocol that foresees the collection of data on pain also in the intensive care unit when possible.

Finally, the study can be improved by comparing the intervention group with an “attention placebo” group that receives “contextual healing” (Guidi et al., 2018) in terms of psychoeducation or psychological information instead of “usual care,” as suggested by Bellani (2008). Using this strategy, it will also be possible to administer follow-up questionnaires at T2 to the control group, allowing an appropriate comparison in terms of timing. In our study, we compared T1 usual care results with T2 intervention group outcomes on perceived self-efficacy and anxiety, assuming that patients in the control group did not change their status on these variables just 1 h after their first collection at T1. This is an assumption that needs to be proven.

## Data Availability Statement

Data and psychological intervention protocol are available from the corresponding author upon reasonable request.

## Ethics Statement

The studies involving human participants were reviewed and approved by Verona Research Ethics Committee (Prog. 1288CESC). The patients/participants provided their written informed consent to participate in this study.

## Author Contributions

VM is the clinical psychologist who delivered the psychological intervention the day before surgery and collected data. OD enrolled and assessed patients at T0 time and collected data. MM is the statistician who conducted statistical and power analysis. ES and DB were data manager. MT and RS are the surgeons who conceived the research. MR conceived the research and contributed to writing the manuscript. CB is the head of the Pancreas Institute and conceived the research. LD conceived the research, devised the psychological intervention protocol, supervised the research and wrote the manuscript. All the authors agreed on the final version of this manuscript.

## Conflict of Interest

The authors declare that the research was conducted in the absence of any commercial or financial relationships that could be construed as a potential conflict of interest.
